# THE PERSISTENCE OF AGE DISCRIMINATION IN SHAPING LATE WORKING LIVES

**DOI:** 10.1093/geroni/igad104.1788

**Published:** 2023-12-21

**Authors:** Rachel Crossdale, Liam Foster, Alan Walker

**Affiliations:** The University of Sheffield, Sheffield, England, United Kingdom; University of Sheffield, Sheffield, England, United Kingdom; University of Sheffield, Sheffield, England, United Kingdom

## Abstract

The aging population and subsequent demographic shift have driven the Extended Working Lives (EWL) agenda to become an important policy goal. However, millions of older workers leave the workforce each year due to unsupportive work environments attributed in part or in whole to ageist policies and practices, raising questions about older workers’ perceptions of themselves in the workforce and what this means for the EWL agenda. Using data from 25 qualitative interviews, our research finds that ageism continues to play a part in the decision-making process of older workers when considering their future. These findings fall into two categories: Age barriers within the workplace and Perceptions. Age barriers within the workplace include tasks or events that incite a feeling of separation from younger peers, such as training, social interaction, and digitization. Perceptions are older workers’ internalized expectations of themselves and their abilities. Our findings suggest these perceptions do not align with the reality for older workers, thus contributing to internalized ageism. The need to target stereotypical attitudes towards older workers was highlighted as an age barrier to employment before the turn of the century. However, despite the Extended working Lives, Active Aging and Fuller Working Lives Agendas changing the policy context around older workers, this research suggests that the same stereotypes continue to pervade older workers both externally from policy and practice and internally in self-perceptions. A life-course approach to policy is needed to improve conditions for older workers and to challenge ageist stereotypes in a meaningful way.

